# Kinesio taping reduces elbow pain during resisted wrist extension in patients with chronic lateral epicondylitis: a randomized, double-blinded, cross-over study

**DOI:** 10.1186/s12891-018-2118-3

**Published:** 2018-06-19

**Authors:** Yen-Ting Cho, Wen-Yen Hsu, Li-Fong Lin, Yen-Nung Lin

**Affiliations:** 1 Department of Physical Medicine and Rehabilitation, Wan Fang Hospital, Taipei Medical University, No.111, Hsing-Long Road, Section 3, Taipei, 116 Taiwan; 2 Department of Physical Medicine and Rehabilitation, Shuang-Ho Hospital, Taipei Medical University, New Taipei City, Taiwan; 3 Graduate Institute of Injury Prevention and Control, Taipei Medical University, Taipei, Taiwan

**Keywords:** Tennis elbow, Physiotherapy, Tape, Epicondylopathy, Tendinopathy

## Abstract

**Background:**

Lateral epicondylitis is frequently seen in racquet sport players and the treatments are usually symptomatic rather than curative. Taping therapy is cheap and easy to apply in the sport field. In this study we valued the effectiveness of Kinesio taping (KT) on immediate pain control for patients with chronic lateral epicondylitis.

**Methods:**

We conducted a randomized, double-blinded, cross-over study with 15 patients with chronic lateral epicondylitis. All participants received two taping sessions in a random order with a 3-day interval in between: one with KT and the other with sham taping (ST). Pain perceived during resisted wrist extension and at rest using numeric rating scale (NRS), the pain-free grip strength, and the pressure pain threshold, were measured before and 15 min after the tape was applied.

**Results:**

A significant reduction of 2.1 ± 1.6 (Z = − 3.081, *P* = 0.002) and 0.7 ± 0.8 (Z = − 2.428, *P* = 0.015) was found on a NRS with KT and ST, respectively, indicating that both taping sessions produced immediate pain relief for resisted wrist extension. Both taping sessions significantly improved the pain-free grip strength with increases of 3.31 ± 5.05 (Z = − 2.615, *P* = 0.009) and 2.43 ± 3.31 (Z = − 2.783, *P* = 0.005) kg found with KT and ST, respectively. Compared with ST, KT exhibited superiority in controlling pain experienced during resisted wrist extension (Z = − 2.168, *P* = 0.030).

**Conclusions:**

Taping produced unneglectable placebo effects on pain relief and painf-free grip strength for patients with lateral epicondylitis, and KT seemed to have additional effects on controlling pain that was elicited by resisted wrist extension.

**Trial registration:**

ISRCTN13618356 (retrospectively registered on 13/02/2017).

## Background

Lateral epicondylitis (tennis elbow) is the most common cause of elbow pain [1], and is commonly seen in racquet sports players with a reported incidence of 9~ 35% and a prevalence of 14~ 41% among tennis players [2]. The dominant upper limb is much more often involved [3]. It typically presents with pain around the lateral epicondyle elicited by forceful wrist extension. This is the result of the degenerative angiofibroblastic hyperplasia of wrist extensor tendons due to repeated microtraumas [4]. Although treatments are usually non-surgical (e.g., oral medications, steroid injections, and physiotherapy), many of them lack sufficient evidence of beneficial effects [5–7]. In some cases, the recovery phase can be as long as several months [8], potentially impacting the quality of life and sports performance of affected individuals [9].

Kinesio taping (KT) is widely used to manage various musculoskeletal problems. Invented by the Japanese chiropractor Kenzo Kase in the 1970s, the tape is an elastic woven-cotton strip with heat-sensitive acrylic adhesive and the maximum available tension of about 40–60% its overall length [10]. Numerous effects of KT are hypothesized, including pain reduction, normalizing muscle function, improving proprioceptive feedback, and correcting articular malalignment [11, 12]. Various clinical effects of KT have been reviewed in a diversity of conditions and populations [13–18]. Various quality and methodology of the trials has influenced the consistency of results in these reviews. The results has been interpreted as either trivial [13] or no effects [14] on muscle strength in healthy adults. KT may [17, 18] or may not [16] reduce pain in the short-term use when compared with minimal treatment, and not be superior when compared with other interventions [17, 18] in patients with musculoskeletal disorders. Despite the inconsistencies, some randomized controlled trials have reported that the KT is beneficial in controlling pain in certain conditions such as acute [19] and chronic low-back pain [20], cervical whiplash [21], and knee pain after joint replacement [22]. Certainly, well-designed research is warranted so that the practitioners can be confident that KT is beneficial for their patients.

The effectiveness of KT in managing lateral epicondylitis has not been adequately explored. In a non-control study with before-after design, Dilek et al. reported that patients’ pain and grip strength significantly improved after applying KT [23]. However, without a control group, those positive findings can be due to the placebo effect. We therefore designed this study with a placebo control to investigate the effectiveness of KT on pain relief. We also focused on the immediate effects on pain reduction during dynamic motions in hope of applying the results to the sport fields. Considering that pain measures are usually subjective and might have great inter-individual variability, we used a randomized cross-over design with self-comparator to maximize statistical power from our sample size. We hypothesized that KT could provide immediate effects on pain reduction in patients with lateral epicondylitis.

## Methods

### Participants

We screened for eligible patients from the rehabilitation outpatient department of Wan-Fang Hospital with a diagnosis of chronic lateral epicondylitis. The criteria for the diagnosis of chronic lateral epicondylitis was based on the clinical presentation and included: (1) typical pain over the lateral epicondyle elicited by resisted wrist extension; (2) tenderness at the lateral epicondyle; and (3) symptoms lasting for at least 2 months. Patients were excluded if they previously had had experience with KT treatment, had had a steroid injection for lateral epicondylitis within the past 3 months, were suspected of having elbow arthritis, or had a wound where the taping was to be applied. If pain was reported in both elbows, the one with more-severe pain was used in our experiment.

### Design

**Fig. 1:**
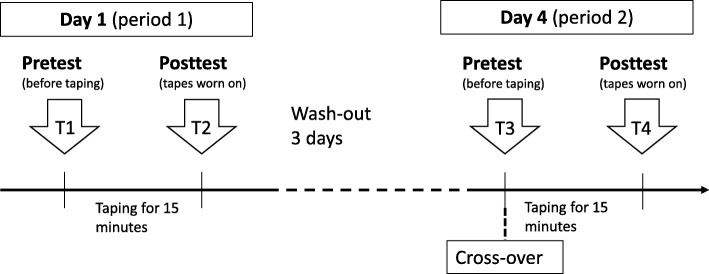
Study process

Participants who met the enrollment criteria and completed the consent form were assigned to receive two taping sessions with different tapes: one with KT and the other one with sham taping (ST). A 2 (treatment) × 2 (period) crossover design was used (Fig. 1). Participants received the two taping sessions in a random order with a 3-day washout interval in between. The physiatrist performing the taping conducted the randomization by coin toss. Oral medications (e.g., nonsteroid anti-inflammatory agents and acetaminophen) and physiotherapy were not allowed since 2 days before the pretest. The Institutional Review Board of Taipei Medical University reviewed and approved the research protocol in the spirit of the *Helsinki Declaration*.

### Taping procedure

**Fig. 2:**
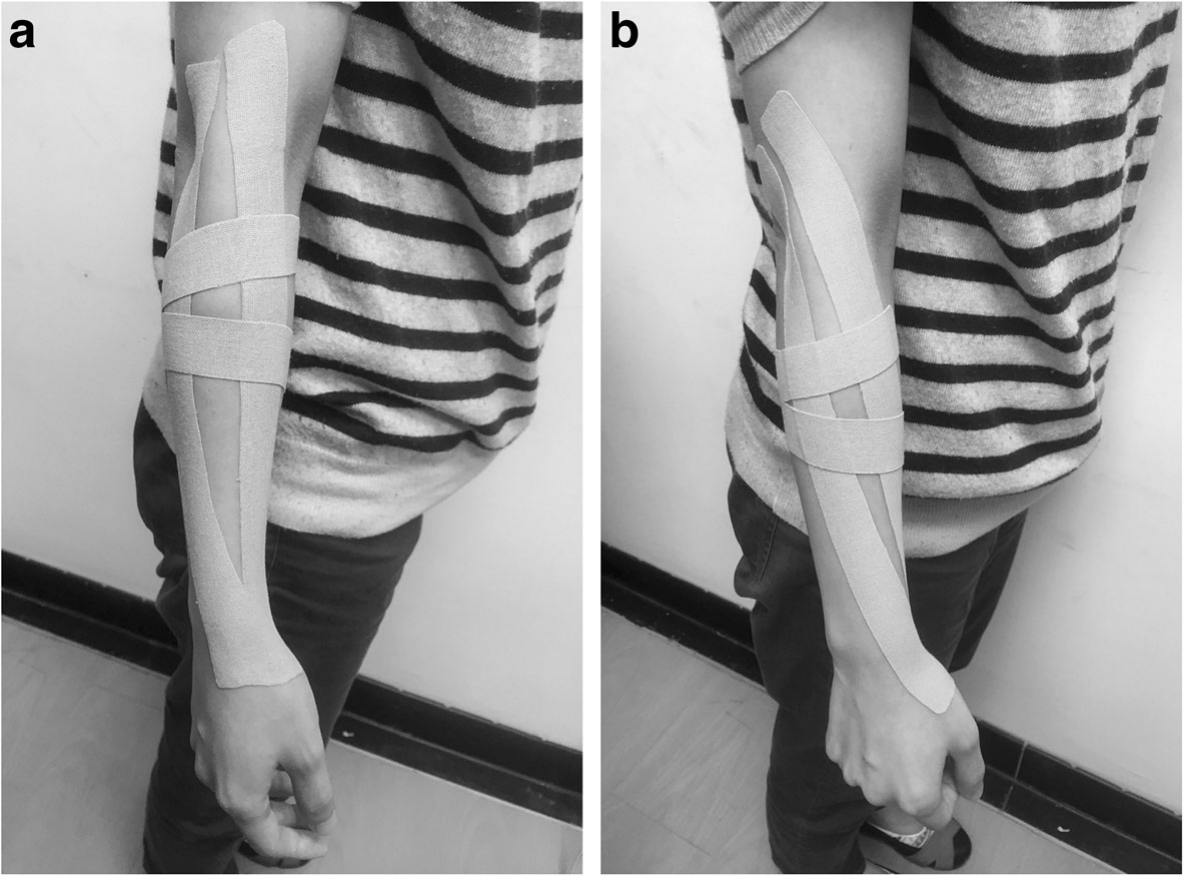
Application of Kinesio taping (KT) and sham taping (ST) for lateral epicondylitis in this study. **a** Kinesio taping. **b** Sham taping with Elastic Adhesive Tapes (3 M™). Note that the soft tissue is bulging between the tape tails from being squeezed by the tension (**a**), and the skin is completely smooth between the tails (**b**). It was difficult to differentiate between the two tape types by their appearance

The Kinesio Tex Tape was used for KT and the Elastic Adhesive Tape (3 M™) for ST. The color and width of the two tapes were very similar, appearance-wise making it hard to differentiate between them (Fig. 2). The taping was performed by an experienced physical therapist. We used two Y-shaped Kinesio strips for the KT procedure for lateral epicondylitis as proposed by Kaze et al. [10], with the main strip applied along the extensor muscles and the second strip vertical to the first one on the proximal forearm (Fig. 2). The main strip was used to inhibit the targeted muscles while the second strip correct the fascia. The patient was positioned with the elbow extended and the wrist ulnar deviated and flexed. After cutting the tape into a Y-shape, we applied the tape head (anchor) of the first strip to the wrist, stretched the tape slightly with approximately 30% of available tension to the tails, laid down the tape ends with no tension, and applied pressure to the tape surface to initiate adhesion. We then applied the anchor of the second strip 1 in. distal and anterio-medial to the lateral epicondyle with no tension, applied approximately 30% tension to each tail across the wrist extensors, laid down the ends at the border of ulnar with no tension, and applied pressure to the tape surface to initiate adhesion. The applied tension was estimated according to the length stretched. For example, if a segment of tape is 4 cm in length under no tension and can be stretched up to 6 cm, the tension when stretched to 5 cm would be (5–4) / (6–4) = 50%. The ST procedure was very similar to that of KT except that the wrist was placed in a neutral rather than a flexed position when applying the tape. ST was also carefully applied tension-free during the entire procedure.

### Basic information

Basic participant characteristics, such as gender, age, duration of disease and affected side were obtained through a short interview with a structured questionnaire including the Patient-Rated Tennis Elbow Evaluation (PRTEE). PRTEE (0~ 100) assessed the pain and disabilities caused by lateral epicondylitis with a higher score indicating more significant impacts [24].

### Outcome measurements

**Fig. 3:**
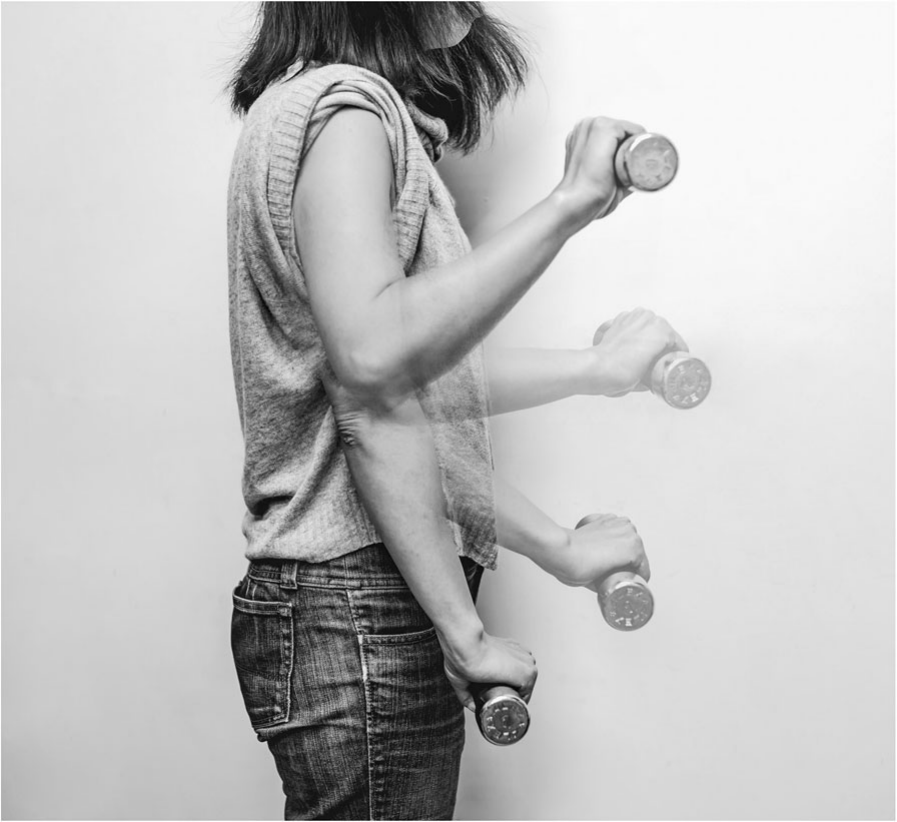
The pain-1 kg test. During the test, participants lifted the weight while flexing the elbow from 0 to 120 degrees and returned the weight to starting position. The wrist was kept pronated during this process so that the wrist extensor muscles were isometrically contracted to counter gravity throughout the process

The primary outcome was the pain experienced during resisted wrist extension, as it had been in other lateral epicondylitis trials [25]. We standardized the pain measuring procedure by having the participant hold an 1-kg weight in a standing posture with the arm relaxed (pain-1 kg). The participant was then asked to slowly lift the weight while flexing the elbow from 0 to 120 degrees and then slowly returning to the starting position (Fig. 3). The wrist was kept pronated during the process to ensure that the wrist extensor muscles were isometrically contracted to counter gravity. The participant repeated the process three times and then reported the degree of perceived pain during the process using an 11-point numerical rating scale (NRS) [26] with 0 = “no pain” and 10 = “worst possible pain”. In a pre-study test with 5 participants, we found the test-retest reliability was good with an intra-class correlation coefficient of 0.91. A reduction of 2 points is thought to be the minimal clinically important difference (MCID) [26].

Other outcomes of interest included pain at rest using the NRS (pain-rest), the pain-free grip strength, (PFG) and the pressure pain threshold (PPT). When measuring the PFG, the subject stood with the elbow in complete extension and the shoulder and radioulnar joints in neutral rotation. The subject then began to squeeze a dynamometer (JAMAR Plus, Patterson Medical, Canada) with increasing force until he/she felt elbow pain [27]. The PPT (i.e., minimum amount of pressure that triggered pain) was quantified by applying the 1-cm^2^ rubber probe tip of a digital algometer (Force Ten FDX Force Gage, Wagner Instruments, USA) to the most palpably tender site over the lateral epicondyle [28]. The PFG and PPT were measured three times and average values used for analysis. An assessor blinded to the treatment assignment performed all measurements before taping (pretest) and 15 min after taping with the tape in situ (posttest). We removed tapes after the posttest. Despite the similar appearances of the KT and ST, participants were asked to wear a long-sleeved shirt to cover the taping so that the assessor was sufficiently blinded to the taping type at the posttest.

### Data analysis

We used non-parametric tests for this small-sample study. Carryover effects were investigated by comparing the outcomes at T1 and T3 with Wilcoxon signed-rank test. The changes caused by taping were assessed using Wilcoxon signed-rank test. To compare the effects between the two taping, we divided the participants by sequence as KT-ST and ST-KT groups. We defined the differences between the two periods for KT-ST group as μ(KT-ST) = μ(KT)- μ(ST); and for ST-KT group as μ(ST-KT) = μ(ST)- μ(KT). Then we compared the μ(KT-ST) and μ(ST-KT) using Mann-Whitney U test, with the H_0_: μ(KT-ST)- μ(ST-KT) = 0, which is equal to test the H_0_: μKT - μST = 0. We also tested the sequence effects by comparing “μ(KT) + μ(ST)” between the two groups. A two-tailed *P* < 0.05 represented the level of significance. Based on a previous KT study [21] that showed an effect size of 0.9 on visual analog scale for pain, a total sample size of 15 participants in this study would provide a power of 0.9 to detect the between-group difference with an ⍺level of 0.05. All analyses were performed using the SPSS statistical package version 17.0.

## Results

Table 1 showed the characteristics of the participants. Fifteen participants who completed the study had a mean age of 52.3 years (SD 8.8) and a disease duration of 4.9 months (SD 2.7). Twelve right hands and three left hands were tested. The PRTEE questionnaire ranged from 24~ 78 with a mean of 46.3 (SD 17.5), indicating a wide range of severity of pain symptoms and disabilities that were caused by lateral epicondylitis.

**Table 1:** Basic characteristics of participants

No.	Gender (M/F)	Age (y)	Duration of disase (months)	Affected elbow (R/L)	PRTEE -pain	PRTEE -ADL	PRTEE -total	Treatment order
1	F	5X	6	R	36	37.5	73.5	ST-KT
2	M	5X	5	R	15	9.5	24.5	KT-ST
3	F	7X	9	R	36	21	57	ST-KT
4	F	4X	3	R	22	13	35	KT-ST
5	F	5X	4	R	25	17	42	KT-ST
6	F	5X	2	L	25	25.5	50.5	ST-KT
7	F	4X	6	L	38	40	78	KT-ST
8	M	6X	4	R	23	23.5	46.5	KT-ST
9	F	5X	5	R	28	12.5	40.5	KT-ST
10	M	4X	3	L	15	15.5	30.5	ST-KT
11	M	4X	2	R	42	33.5	75.5	KT-ST
12	F	6X	3	R	22	20	42	ST-KT
13	M	6X	3	R	19	20	39	KT-ST
14	F	4X	6	R	15	9	24	ST-KT
15	F	4X	12	R	29	7.5	36.5	KT-ST

*M* male, *F* female, *BMI* body mass index, *R* right, *L* left, *PRTEE* patient-rated tennis elbow evaluation, *ADL* activity of daily living, *KT* Kinesio taping, *ST* sham taping

No significant differences existed between T1 and T3 regarding all the outcomes, with a Z value of − 1.540, − 1.469, − 1.051, − 0.511 for pain-1 kg, pain-rest, PFG, and PPT respectively (all *P* > 0.05, not shown in Tables), indicating no significant carryover/period effects. The pretest, posttest, and changes in outcome parameters regarding the taping types were shown in Table 2. After taping, both taping sessions significantly produced immediate pain relief during resisted wrist extension (*P* = 0.002 and 0.015 for KT and ST respectively) and increases in pain-free grip strength (*P* = 0.009 and 0.005 for KT and ST respectively). In addition, the ST significantly decreased the pain at rest (*P* = 0.014) and the KT increased the PPT (*P* = 0.016).

**Table 2:** Means (SD) for pretest, posttest, and changes in outcomes by tapings

	Pretest	Posttest	Difference	^a^Significance for the changes	
			(Posttest - pretest)	Z-value	*P*-value
Pain-1 kg (0~ 10)					
Kinesio taping	4.4 (2.4)	2.3 (2.0)	−2.1 (1.6)	−3.081	0.002
Sham taping	3.3 (2.3)	2.7 (2.1)	−0.7 (0.8)	− 2.428	0.015
Pain-at rest (0~ 10)					
Kinesio taping	1.7 (2.1)	1.0 (1.3)	−0.7 (1.5)	−1.633	0.102
Sham taping	2.1 (2.1)	1.5 (1.6)	−0.6 (0.7)	−2.460	0.014
PFG (kg)					
Kinesio taping	10.70 (8.03)	14.02 (10.56)	3.31 (5.05)	−2.615	0.009
Sham taping	12.59 (8.44)	15.01 (10.47)	2.43 (3.31)	−2.783	0.005
PPT(lbf)					
Kinesio taping	3.1 (2.6)	3.9 (4.1)	0.8 (1.6)	−2.414	0.016
Sham taping	2.5 (1.7)	3.5 (4.1)	1.0 (3.2)	−1.162	0.245

*Pain-1 kg* pain when holding a 1-kg weight, *PFG* pain-free grip strength, *PPT* pressure pain threshold

^a^Significance was assessed with Wilcoxon signed-rank test

The results of comparison for taping and sequence effects were shown in Table 3. Significant between-taping difference was noted in pain-1 kg (*P* = 0.030), indicating the KT was superior to ST in controlling the pain experienced during resisted wrist extension. No significant differences were found regarding the other parameters, and no significant sequence effect was found in the pain parameters (all *P* > 0.05).

**Table 3:** Between-group differences regarding the outcome measurements

	^a^Effect at	^b^Effect at	Comparison for taping effects			Comparison for sequence effects		
	Period 1	Period 2	Period 1-Period 2	Z-value	^c^*P*-value	Period 1+ Period 2	Z-value	^c^*P*-value
Pain-1 kg (0~ 10)								
KT-ST (*n* = 9)	−1.9 (1.4)	−0.8 (1.0)	− 1.1 (1.8)	−2.168	0.030	−2.7 (1.5)	−0.240	0.811
ST-KT (*n* = 6)	−0.5 (0.5)	−2.3 (2.1)	1.8 (2.2)			−2.8 (2.0)		
Pain-at rest (0~ 10)								
KT-ST (*n* = 9)	−1.0 (1.7)	−0.9 (0.8)	−0.1 (1.8)	0	1.000	−1.9 (2.0)	−1.538	0.124
ST-KT (*n* = 6)	−0.2 (0.4)	−0.5 (0.8)	0.3 (0.5)			−0.7 (1.2)		
PFG (kg)								
KT-ST (*n* = 9)	3.3 (6.3)	2.9 (3.4)	0.4 (5.3)	−0.589	0.556	6.2 (8.7)	−0.059	0.953
ST-KT (*n* = 6)	1.7 (3.3)	3.3 (2.8)	−1.6 (2.2)			5.0 (5.7)		
PPT(lbf)								
KT-ST (*n* = 9)	0.6 (0.5)	0.1 (0.6)	0.5 (0.4)	−0.354	0.723	0.6 (1.0)	−0.707	0.480
ST-KT (*n* = 6)	2.3 (4.9)	1.2 (2.6)	1.1 (2.5)			3.5 (7.5)		

*Pain-1 kg* pain when holding a 1-kg weight, *PFG* pain-free grip strength, *PPT* pressure pain threshold

^a^Effect at Period 1 = Posttest (T2)- Pretest (T1)

^b^Effect at Period 2 = Posttest (T4)- Pretest (T3)

^c^Significance was assessed with Mann-Whitney U test

## Discussion

In this study, we explored the effects of KT on pain relief by measuring several pain parameters in patients with lateral epicondylitis. Our results showed that both taping sessions (KT and ST) produced significant improvement in pain experienced during resisted wrist extension and pain-free grip strength. However, KT was superior to ST in reducing pain elicited by resisted wrist extension, while producing an average reduction of 2.1 points on the NRS, reaching the MCID. Our results supported the use of KT as a temporary pain management for lateral epicondylitis.

Lateral epicondylitis is commonly seen in racquet sports. Substantial eccentric contractions of the extensor carpi during the backhand stroke are likely the cause of repetitive microtrauma leading to the lateral epicondylitis [29]. Lateral epicondylitis also has a high prevalence among the general population, affecting about 1~ 3% of people of working age [30]. Traditionally, the management usually relies on conservative treatments, such as oral non-steroidal anti-inflammatory drugs, physical agents (eg, ultrasound, electrical stimulation), therapeutic exercise, or steroid injection. Even with these treatments, patients usually have to endure symptoms for several months. Therefore, an effective temporary management, such as taping, can potentially improve the quality of life and sport performance.

As shown in Table 2, KT significantly improved the pain-1 kg, PFG, and PPT, yet the mechanism is unclear. The initial concept of applying KT when introduced is to reduce the build-up of fluid between and within the layers of the soft tissue [10]. However, the correlation between this concept and the effects on pain relief is not well explained. Therefore, some other mechanisms for pain relief have been hypothesized. For one, it was suggested that non-neuronal cells may act as a key signaling pathway for sensory modalities by triggering adjacent nerve terminals [31]. As we understand, somatic pain is perceived when noxious stimuli activate specific receptors (nociceptors) of thinly myelinated Aδ- and unmyelinated C-fibers. Some studies suggested that keratinocytes may represent non-neuronal primary transducers of mechanical stimuli, probably via a signal transduction cascade mechanism, to evoke a response in adjacent C-fibers [31, 32]. If so, cutaneous stretching produced by KT may possibly affect pain processing via keratinocytes. Furthermore, stimulation by cutaneous stretching may also interfere with the transmission of pain by facilitating a pain inhibitory mechanism. By gate control theory, the constant somatosensory input by cutaneous stretching could potentially close the “gates” to painful input, which prevents pain sensation from traveling to the central nervous system [33].

Another possible explanation is related to muscle activities modulation. Several studies suggested that KT can potentially modulate muscle activities [34–36]. A study conducted by Hsu et al. revealed that the muscle activity was decreased in lower trapezius but increased in serratus anterior and upper trapezius after taping on the lower trapezius [34]. Wong et al. and Yeung et al. also found that taping on the vastus medialis shortened the time to generate peak torque of knee extension [35, 36]. These preliminary reports may lead to the hypothesis that KT reduces pain through modulating muscle activities, possibly accounting for why KT was superior to the ST in reducing pain during resisted wrist extension but not at rest. Therefore, we speculate that the benefits of KT may partly come from the decreased load on the lateral epicondyle during the contraction of wrist extensors. For example, the main strip applied parallel to the forearm may inhibit the muscle activity with its elasticity and reduce the irritation of the enthesis. Meanwhile, it is our hypothesis that the second strip applied vertical to the forearm may act in a similar way to the commonly used elbow brace which produces a wider muscle origin thereby decreasing the stress at the lateral epicondyle [27]. However, further studies are needed to explore the possible mechanisms.

It is interesting to note that ST also exhibited significant improvements in pain-1 kg and the PFG (Table 2). In our study, we used elastic tape for ST, which might have had some effects when the tape was stretched and a traction force was created. However, the treatment effect should have been minimal, as we carefully avoided any tension when applying ST over the forearm. Therefore, those improvements may be attributed to the placebo effect.

Our findings were similar to a recently published study conducted by Shakeri et al. [37] The authors designed a 4-day intervention to compare the KT with the placebo (KT without tension) and evaluate the effectiveness for patients with lateral epicondylitis. Both tapings resulted in reduced pain during activities and the degree of arm disabilities after 4 days of intervention but KT exhibited significantly greater improvement. The authors also found no difference between the KT and placebo groups regarding the grip strength and pain threshold. Different from Shakeri’s work, our study was intended to understand the potential benefits of applying the KT in the sport field. This is the reason we measured the immediate effects on dynamic motion by lifting a 1-kg weight to simulate the backhand stroke of racquet sports.

In contrast, a Chinese study group published a series of researches recently and provided negative evidence. They conducted two crossover studies to assess the effects of KT applied to wrist extensors at different taping conditions (eg, facilitatory KT, inhibitory KT, no KT) among healthy adults [38] and patients with lateral epicondylitis [39]. The authors found no difference between these different conditions regarding maximal grip strength and electromyographic activities in both populations [38, 39]. They also found no significant effects on pain intensity and PFG, in patients with lateral epicondylitis [39]. While the authors reported null effects, they did not consider the carryover and sequence effects which are essential methodological issues for a crossover study, so that the interference between the conditions could possibly lead to the negative results. Considering that KT is a cheap and convenient option that can potentially manage pain instantly, further exploration of its treatment mechanism and effects with well-designed researches is certainly worthwhile.

Several study limitations should be addressed. First, the sample size was small. Second, only the immediate effects of KT were evaluated. Whether these effects lasted beyond 15 min is unknown. Third, we only explored the effects on pain elicited by isometric wrist extension with 1-kg resistance, so whether KT is effective at higher resistance is also unknown. These issues are important to consider before applying taping in the sport field. Fourth, placebo effect of any kind of taping that the subjects might expect benefit on that cannot be ruled out. And finally, although we would like to project our results onto people who sustain lateral epicondylitis from sport injuries, we did not specifically limit our patient selection to athletes.

## Conclusions

Taping produced unneglectable placebo effects on pain relief and PFG for patients with lateral epicondylitis, and KT seemed to have additional effects on controlling pain that was elicited by resisted wrist extension.

## Abbreviations

KT: Kinesio taping
MCID: Minimal clinically important difference
NRS: Numerical rating scale
PFG: Pain-free grip strength
PPT: Pressure pain threshold
PRTEE: Patient-rated tennis elbow evaluation
ST: Sham taping
